# Bedside Scoring System for Predicting Adverse Outcomes Among Patients Suffering From SARS-CoV-2 Infection

**DOI:** 10.7759/cureus.32009

**Published:** 2022-11-29

**Authors:** Shravanthi Woodayagiri, Swathy Moorthy, Emmanuel Bhaskar, Lakshmi Marappa

**Affiliations:** 1 General Medicine, Sri Ramachandra Institute of Higher Education and Research, Chennai, IND; 2 Internal Medicine, Sri Ramachandra Institute of Higher Education and Research, Chennai, IND

**Keywords:** covid-19 retro, diabetes, glycemic level, severity scoring, influenza-like illness, sars-cov-2

## Abstract

Aim

To develop a clinical risk score to predict adverse outcomes among diabetic hospitalized COVID‐19 patients

Methods

The data was collected retrospectively from patients hospitalized with the SARS-CoV-2 virus at Sri Ramachandra Institute of Higher education and research. It integrated independent variables such as sex, age, glycemic status, socioeconomic status, and preexisting lung conditions. Each variable was assigned a value and the final score was calculated as a sum of all the variables. The final score was then compared with patient outcomes. The patients were scored from 0 to 8 and a score of 3 or more was considered as being at greater risk for developing complications. Number of mortalities in each group, any clinical deterioration requiring ICU admission, and the number of patients requiring a prolonged hospital stay of more than 10 days in each group were noted and the results compared.

Results

Higher blood glucose levels and preexisting lung conditions like chronic obstructive pulmonary disease (COPD), asthma, and pulmonary tuberculosis have been associated with a higher risk of developing complications related to SARS-CoV-2 illness. Of the 5023 patients enrolled in the study, 2402 had a score of 2 or below, and 2621 had a score of 3 or above. Among patients with a score of 2 or below 1.7% of the patients contracted a severe disease resulting in death. 2.9% were shifted to ICU, but recovered and 12.2% of patients had a prolonged hospital stay. Of those with a score of 3 or greater, 5.1% died, 7.36% were shifted to ICU, but recovered, and 19.5% required a prolonged hospital stay. The observed results were analyzed using the Chi-square test and were found to be significant at a p-level of 0.0001.

Conclusion

This clinical risk score has been built with routinely available data to help predict adverse outcomes in diabetic patients hospitalized with the SARS-CoV-2 virus. It is a good tool for resource-limited areas as it uses readily available data. It can also be used for other severe acute respiratory illnesses or influenza-like illnesses.

## Introduction

Global pandemics have occurred periodically through time immemorial. The SARS-CoV-2 pandemic of the 21st century has been one of the worst pandemics faced in recent times. To date, there have been over 500 million positive cases and close to 6.5 million deaths worldwide [1].

Several studies have demonstrated that admission hyperglycemia was associated with an increase in poor outcomes and mortality in hospitalized patients presenting with an infectious disease. It is known that type 2 diabetes is an independent risk factor for contracting a more severe form of SARS-CoV-2 infection [2-6]. This study aims to integrate admission random blood sugars with other independent variables to create a scoring system to predict worse outcomes in patients suffering from the SARS-CoV-2 virus.

## Materials and methods

The data was collected retrospectively from patients hospitalized with the SARS-CoV-2 virus at Sri Ramachandra Institute of Higher education and research between March 2020 and March 2022.

Inclusion criteria

Patients >18 years who have been diagnosed with SARS-CoV-2 infection; type 2 diabetes and are on treatment; impaired glucose tolerant patients; stress-induced hyperglycemia

Exclusion criteria

Patients <18 years of age with SARS-CoV-2 infection; pregnancy; patients with chronic illness such as malignancies, autoimmune disease, chronic diseases requiring steroids, smokers

After obtaining informed consent, a detailed study proforma was filled and the demographic details such as age, sex, and socioeconomic background were noted. Further, the patient’s symptoms, comorbidities, and random blood sugar on admission were also noted. The patient’s clinical course was followed and any complication or adverse events, including the need for intensive care was documented till discharge.

The scoring system integrated independent variables such as glycemia, sex, socioeconomic status, and preexisting lung conditions. Each variable was assigned a value and the final score was calculated as a sum of all the variables. This scale has been adapted from a study done in Kuwait by Alhamar et al. [7]. Each individual parameter was chosen based on preexisting knowledge of independent variables known to result in poorer outcomes among SARS-CoV-2-infected patients. The parameters and individual scores are listed in Table 1.

**Table 1:** Individual score for each clinical parameter

Criteria	Score
Male	1
Low socioeconomic status	1
Age >60 years	1
Age >80 years	2
Random blood glucose 126-199 mg/dl	2
Random blood glucose >200 mg/dl	3
Preexisting lung condition	1

The final score was then compared with patient outcomes and the number of deaths in each group was calculated as the primary endpoint. Clinical deterioration resulting in a shift to an ICU or prolonged hospital stay of more than 10 days (marker of morbidity) was noted as the secondary endpoint.

## Results

A total of 5023 patients were enrolled in the study. The demographic details of the patients are mentioned below in Table 2.

**Table 2:** Demographic details of patients enrolled in the study

Parameter	Distribution	
Sex	Male	3048
	Female	1975
Age	<59	3369
	60 to 79	1473
	>80	181
Socioeconomic status	Low	2527
	High	2496
Blood glucose levels (at admission)	<125 mg/dl	2467
	126 – 199 mg/dl	1495
	>200 mg/dl	1061

Each patient was assigned a score, which was then compared with the primary and secondary endpoint, and the results are tabulated in Table 3.

**Table 3:** Number of patients satisfying the primary and secondary endpoints per score

Outcomes		Final score								
Endpoint	0	1	2	3	4	5	6	7		
	No of patients satisfying each endpoint								Total	
Morbidity	-	117	177	169	173	142	28	1	807	
Mortality	-	19	23	25	31	55	23	-	176	
ICU requirement	-	29	41	63	63	57	8	2	263	
Total	260	1001	1141	949	910	633	118	11	5023	

**Figure 1:**
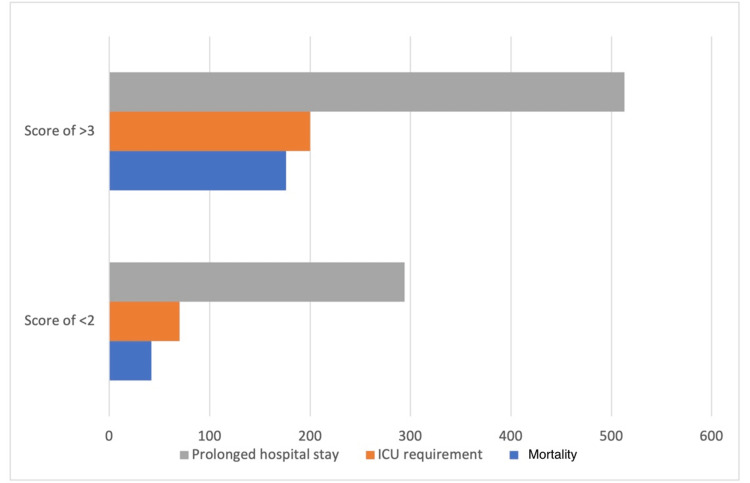
Distribution of patients satisfying primary and secondary endpoints among low- and high-score groups

The patients were then divided into those who have a score of 2 or below (n = 2402) or 3 and above (n = 2621). Table 4 and Figure 1 show the distribution of various endpoints within each category.

**Table 4:** Comparison of the number of patients satisfying the primary and secondary endpoints having a low and high score, respectively

	Final score		
Endpoint	0-2	3-7	
	No of patients satisfying each endpoint		
Morbidity	294	513	807
Mortality	42	134	176
ICU requirement	70	193	263
Total	2402	2621	5023

Of the patients with a score of 2 or below, 1.7% (42/2402) patients contracted a severe disease resulting in death, 2.9% (70/2402) were shifted to ICU and recovered, and 12.2% (294/2402) patients had a prolonged hospital stay of more than 10 days.

Of those with a score of 3 or greater 5.1% (134/2621) patients satisfied the primary endpoint of the patient contracting a severe form of the disease which resulted in death. 7.36% (193/2621) satisfied the secondary endpoint of requiring ICU admission and 19.5% (513/2621) patients had a prolonged hospital stay of more than 10 days. The observed results were analyzed using the Chi-square test and were found to be significant at a p-level of 0.0001.

## Discussion

Over the course of the pandemic, many studies have been done to identify various factors that may predict worse outcomes in patients testing positive for the SARS-CoV-2 virus. As with most diseases, elderly individuals were found to be more susceptible and were found to have a poorer prognosis when compared to younger individuals [8,9]. In this study, 1.6% of people under 60, 6.4% of people between the ages of 60 and 79, and 13.8% of patients admitted with SARS-COV-2 above the age of 80 had severe disease, resulting in death. This is in accordance with previously done studies.

Due to sub-par living conditions, people belonging to lower socioeconomic strata were believed to be more susceptible to the disease [10]. This is partially due to rapid transmission of the virus, overcrowding resulting in increased viral load, and also due to the lack of hygienic living conditions of the aforementioned group. In our study population, the number of patients admitted belonging to lower socioeconomic strata was more, this may indicate more transmissibility of the virus among persons belonging to lower economic strata when compared to those from a more affluent background. The present study too noted a modest increase in mortalities between the two groups, 4.3% and 2.6%, respectively, however, due to the gross difference in number of patients belonging to each group, these results were not found to be so significant.

SARS-CoV-2 is primarily a respiratory virus and enters into the alveoli after aerosol inhalation via binding to the angiotensin-converting enzyme 2 (ACE2), on the surface of host cells [11]. Hence, any preexisting lung conditions such as asthmatics, patients with obstructive airway disease, or fibrosis due to various pathologies were at greater risk for a severe form of the disease. This study showed no significant difference in mortality between the two groups. This may be due to the incongruity between the number of patients belonging to each group.

A meta-analysis published by Abate et al. showed an increased incidence of severe disease in males as compared to females [12]. They attributed this to males being more likely to partake in activities such as smoking or alcohol consumption as well as increased occupational exposure to smoke and harmful chemicals. However, some studies done in mice have shown that estrogen levels in females may suppress cytokine release and reduce the incidence of complications arising from the virus [13]. This study showed similar results with 3.7% of males and 3.1% of females having died.

Fadini et al. conducted a study in which 413 subjects were enrolled. One hundred and seven of whom (25.6%) had diabetes, including 21 newly diagnosed. Adverse outcomes, resulting in shift to an ICU or death occurred in 37.4% of patients with diabetes compared to 20.3% in those without (p < 0.001). Admission glucose was correlated with the clinical condition and it was observed that adverse outcomes were mostly mediated by a worse respiratory function. It was observed that hyperglycemia was a powerful predictor of COVID-19 severity due to rapid respiratory deterioration [14]. The present study too showed similar results with 118/1061 of patients with greater than 200 mg/dl blood sugar on admission, 156/1495 of patients presenting with blood sugar between 126 and 199 mg/dl, and 139/2467 of patients presenting with normal random blood sugar, of which 72 of the 1061 (6.7%), 57 of the 1495 (3.8%) and 47 of the 2467 (1.9%) succumbed to the infection.

Possible mechanisms for this increased mortality include hyperglycemia-induced changes in coagulation, worsening of endothelial function, and inflammatory cytokine overproduction. A study done by Tiwari et al. examined the effects of hyperglycemia on the inflammatory response of patients with sepsis. Higher levels of inflammatory cytokines such as TNF-alpha were seen in diabetics with uncontrolled sugars. The duration of TNF-alpha expression was also found to be prolonged, leading to more persistent inflammation and tissue damage. Cytokine dysregulation associated with hyperglycemia was suspected to be one of the factors responsible for more severe infection seen in diabetics compared with normal individuals [15].

The score was adapted from a similar study done in Kuwait in 2022 [7]. The study considered various independent prognostic indicators to predict the occurrence of complications or mortalities related to the SARS-CoV-2 virus. The index study aimed at integrating more non-variable parameters such as age to increase the predictive value and consistency of the scoring system.

## Conclusions

Patients with a higher score were found to have a higher mortality rate and were at risk of developing more severe diseases, requiring ICU care or prolonged hospital stays. The scoring system is an easy-to-use bedside tool. It can easily be used in resource-limited settings as it integrates readily available demographic data with a single blood sugar value at the time of presentation and can be used to predict adverse outcomes in patients suffering from severe acute respiratory illness or influenza-like illnesses.
